# Epidemiology and serological detection of Rift Valley Fever disease in farm animals in southern Egypt

**DOI:** 10.4102/ojvr.v88i1.1877

**Published:** 2021-02-03

**Authors:** Hassan Y.A.H. Mahmoud, Alsagher O. Ali

**Affiliations:** 1 Department of Animal Medicine, Division of Infectious Disease, Faculty of Veterinary Medicine, South Valley University, Qena, Egypt

**Keywords:** farm animals, RVFV, southern Egypt, epidemiology, camel, infectious diseases

## Abstract

In this study, the serological surveillance of Rift Valley Fever virus (RVFV) in southern Egypt was carried out for 460 serum samples collected from domestic animals (unvaccinated), including cattle, sheep, goat, camel and donkey reared in three different provinces (Qena, Luxor and Aswan). Enzyme linked immunosorbent assay (ELISA) was used to detect RVFV antibodies. The results showed that 97 out of 460 animals were positive by using blocking ELISA. The percentage of RVFV infection in cattle, sheep, goat, camel and donkey was 5.55%, 65.21%, 14.44%, 20.65% and 0%, respectively. Geographical distribution and breeding system were taken into consideration for RVFV infection in these animals. The most prevalent type of infection was identified in intensive breeding farms systems (27.63%), and then in individual breeding systems (11.68%). Qena had a higher infection rate of RVFV (23.55%), in comparison to Aswan and Luxor (20.65% and 14.14%, respectively). Marked seroprevalence recorded in this study indicates a high incidence of infection in sheep (65.21%) and camel (20.65%); this necessitates the application of more effective strategies to control these types of infections in Egypt. This study provides a concise picture about the RVFV disease in southern Egypt. We need more similar studies targeted to clarify the reliable epidemiological status of RVFV disease in southern Egypt and other localities.

## Introduction

Rift Valley Fever (RVF) is a vector-borne viral disease caused by Rift Valley Fever virus (RVFV), a member of the *Bunyaviridae* family and *Phlebovirus* genus that primarily affects domestic ruminants, causing large epizootics with high mortality rates in young animals and abortions in affected dams (Mansfield et al. 2015; Shabani et al. 2015). The virus was discovered in 1930 during an outbreak that affected livestock in East Africa (Daubney & Hudson 1931; Boushab et al. 2015). Six years later, RVFV antibodies were found in human sera from southern Sudan (Findlay et al. 1936). The primary major human epidemic occurred in 1951 in South Africa (Tolou et al. 2009). Since then, multiple outbreaks were reported in several parts of Africa and the Middle East, notably in Egypt (1977, 2003) (Faye et al. 2007; Sissoko et al. 2009), Kenya (1997–1998, 2006–2007) (Faye et al. 2010), Saudi Arabia and Yemen (2000–2001) (Al-Hazmi et al. 2003; Nabeth et al. 2001), Sudan (2007, 2010) (Hassan et al. 2011; Nabeth et al. 2001), Mayotte (2008) (Sissoko et al. 2009) and Mauritania (1987, 1993–1994, 1998, 2003, 2010, 2012) (Boushab et al. 2015; Faye et al. 2007; Nabeth et al. 2001).

The disease occurrence follows the unusual trend of heavy rainfall leading to flooding, resulting in providing a conducive environment for dormant mosquito eggs infected by RVFV to hatch and become predominant mosquito populations that transmit virus to animals and subsequently from animals to humans (Shabani et al. 2015). However, currently, there is no proof for person-to-person transmission of RVFV (Rakotoarivelo et al. 2011; Shabani et al. 2015). In humans, RVFV infection is typically asymptomatic or causes influenza-like illness accompanied by fever and headache but occasionally leads to serious complications, such as haemorrhagic syndromes, retinitis, encephalitis and death (Adam, Karsany & Adam 2010; Al-Hazmi et al. 2003; Mansfield et al. 2015; Shabani et al. 2015).

During the late 1980s, a new extension of the geographic range of RVFV into western Africa was detected. In 2000, RVFV caused an epidemic in Saudi Arabia and Yemen, which was the first time that RVFV was detected outside Africa (Balkhy & Memish 2003; Madani et al. 2003).

Various wildlife species including buffalo and camel have been shown to be seropositive in endemic areas, suggesting a role for these animals in the virus life cycle (Britch et al. 2013).

Rift Valley Fever virus (RVFV) could also be transmitted to different mosquito species that function as linking vectors to other wild and farm animals and to humans, which can cause more amplification of the transmission cycle (Martin et al. 2008).

Rift Valley fever disease (RVFD) is a vector-borne viral disease of domestic ruminants characterised by widespread abortions and infant deaths, and flu-like symptoms. It is endemic in Egypt, Kingdom of Saudi Arabia and Yemen. Outbreak occurs during periods of high downfall or in the environments supporting the proliferation of RVFV-infected dipteran vectors. RVFV is classified as a category (A) priority infective agent by the National Institute of Allergy and Infectious Diseases, showing the possibility to cause social disturbances and requiring public health awareness. RVFV is the third most dangerous animal threat according to the United States Department of Agriculture Animal and Plant Health Inspection Service. Rift Valley fever virus is a sensible danger that spreads to new geographic areas through the movement of virus-carrying vectors confined within aircrafts and ship cargo holds and the supporting ecological conditions. Vector transmission is poorly understood because of lack of information regarding mosquito ecologies, increasing the risk of entrance of RVFV to a new area.

## Materials and methods

### Animals and geographic locations

**FIGURE 1:**
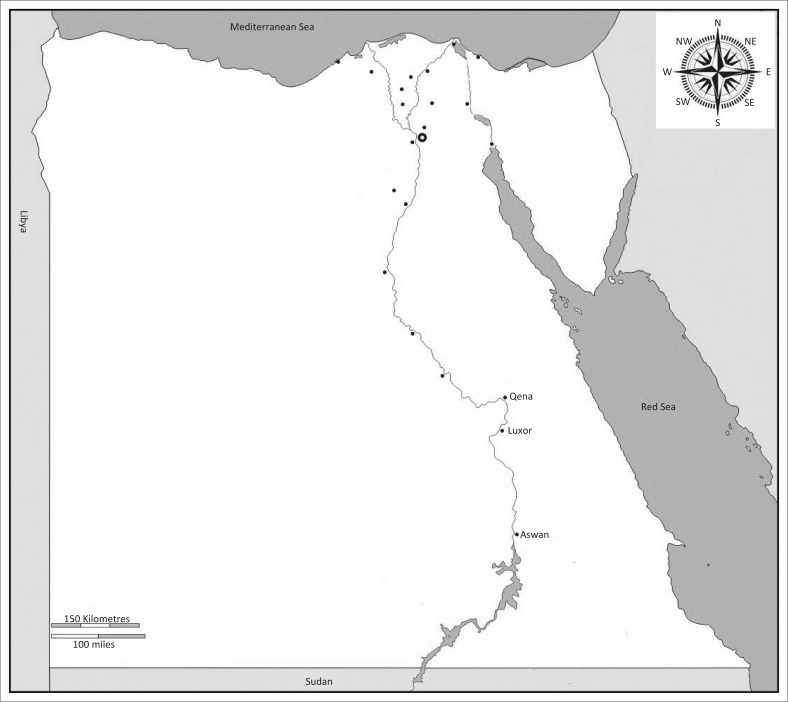
Map of Egypt showing the location of Qena, Luxor and Aswan governates, where the samples were collected.

A total of 460 serum samples were collected from apparently and clinically healthy animals, including cattle, sheep, goat, camel and donkey of different age, sex, breeding system and location. Serum samples were collected from animals during the period from May 2017 to June 2019, randomly in different villages in Aswan, Qena and Luxor governorates in southern Egypt (Figure 1), from individual owners and smallholder farms located in a similar environmental and husbandry conditions, characterised by hot and dry weather. In addition, there were 20 sheep that could not be classified as part of the individual or intensive breeding system.

### Clinical examination

Clinical examination was performed for animals physically, including breed, age and sex; body condition, temperature, respiratory rate and mucous membranes were examined and recorded for individual animals in this study.

### Blood sampling

Blood samples were collected through vein puncture from each animal in glass tubes without anticoagulant, and serum was separated by centrifugation and then stored at −20 °C until use.

## Blocking enzyme-linked immunosorbent assay

Detection of specific antibodies to the N-protein of RVFV antibodies does not depend on the animal species and can therefore be used with sera samples of all species affected by RVFV, according to the manufacturer’s instructions (FVR.K.3/5 Ingezim Rift Valley Fever™ Compac (Immunologiay Genetica Aplicada S.A, Madrid, Spain).

## Technical basis of the test

The recombinant N-protein of the RVFV was used as the antigen, which is used to bind to a polystyrene plate, and after incubating the sera samples, a RVFV-specific monoclonal antibody (Mab) was added. If the sample contains antibodies specific of the virus, they will not allow the binding of the labelled Mab to the antigen, whereas if it does not contain specific antibodies, the Mab will bind to the antigen, which is coating the plate. By washing the plate to eliminate all non-fixed material, the presence or absence of labelled Mab can be detected by adding the substrate, which, in the presence of the peroxidase, will develop a colorimetric reaction. If there has been colour development, it means that the conjugate has bound to the antigen, and therefore the sample is negative. On the other hand, if the sample contains specific antibodies to RVFV, they will block the binding of the conjugate and there will be no colour development.

## Test procedure

Before starting the test, all reagents should be in room temperature (20 °C – 25 °C) for 20 min. After that add 80 µL of the diluent fluid to each well and then add 20 *μ*L of each sample in every well to be assayed. Add 100 *μ*L of positive control (PC) and negative control (NC) to their respective wells, and then seal the plate and incubate for 45 min at 22 °C – 25 °C. Wash the plate for four times and then add 100 *μ*L of the ready conjugate to each well, and seal the plate and incubate for 30 min at 22 °C – 25 °C. Wash the plate for four times and add 100 *μ*L of the substrate solution to each well and keep the plate at room temperature for 10 min, and then add 100 *μ*L of the stop solution to each well and read the optical density (OD) of each well at 405 nm.

## Interpretation of the test

The test is considered valid when the OD of the NC is higher than 0.8, and the PC is lower than 0.25. Calculate the inhibition percentage (IP) of each sample as follows: IP of sample = 100 – ([sample OD/NC OD] × 100), samples will be considered positive when the IP is ≥ 45%, samples will be considered negative when the IP is ≤ 40%, and samples with an IP between 40% and 45% should be considered as uncertain.

### Ethical consideration

The study was reviewed and approved by Research Code of Ethics at the South Valley University (RCOE-SVU).

## Result

Sera samples were collected from 460 animals, including cattle, sheep, goat, camel and donkey in South Egypt from three governorates (Aswan, Luxor and Qena) (Table 1). Investigated sera of animals revealed that the seropositive state of animals against RVFV was 21.08% (97/460), in overall samples, and, that for each species, cattle, sheep, goat, camel and donkey were 5.55% (5/92), 65.21% (60/92), 14.44% (13/92) and 0% (0/92), respectively (Table 2). Epidemiological status was established to clarify the influence of age, sex, location and management system (individual/intensive farming system). It revealed that Qena had a higher infection rate (23.55%) than Aswan and Luxor, 20.65% and 14.14%, respectively (Table 3). Marked infection was recorded in sheep (65.21%) and camels (20.65%), more than goats (14.44%), cattle (5.55%) and donkeys (0.0%).

**TABLE 1:** Classification of animals according to geographical distribution, age, sex and breeding system.

Type of animals	locations	Total number of animals	Age (years)	Sex		Breeding system	
				Male	Female	Individual	Intensive
Cattle	Qena city	92	1–2	12	80	12	80
Sheep	Qena city	92	6 months–2	52	40	22	50
Goat	Luxor city	92	1–2	19	73	20	72
Camels	Aswan city	92	2–3	84	8	8	84
Donkey	Qena city	92	2–3	92	0	92	0
**Total**		**460**	**-**	**259**	**201**	**154**	**286**

**TABLE 2:** Infection rate of Rift Valley Fever in cattle, sheep, goat, camel and donkey.

Species	Positive		Negative		Total
	*n*	%	*n*	%	
Cattle	5	5.55	87	94.45	92
Sheep	60	65.21	32	34.79	92
Goat	13	14.44	79	85.66	92
Camel	19	20.65	73	79.35	92
Donkey	0	0.00	92	100.00	92
**Total**	**97**	**21.08**	**363**	**79.92**	**460**

**TABLE 3:** Rift Valley Fever infection in animals regard to location, age, sex and breeding system.

Factors	Location						Age						Sex				Breeding system			
	Aswan		Luxor		Qena		6 months–1 year		1–2 years		2–3 years		Male		Female		Individual		Intensive	
	*n*	%	*n*	%	*n*	%	*n*	%	*n*	%	*n*	%	*n*	%	*n*	%	*n*	%	*n*	%
Positive number of tested animals	19	20.65	13	14.14	65	23.55	50	54.35	73	39.67	19	10.32	60	23.17	37	18.41	18	11.68	79	27.63
Negative number of tested animals	73	79.35	79	86.86	211	76.45	42	45.65	111	60.33	165	89.68	199	76.83	164	81.59	136	88.32	207	72.37
**Total number of tested animals**	**92**	**-**	**92**	**-**	**276**	**-**	**92**	**-**	**184**	**-**	**184**	**-**	**259**	**-**	**201**	**-**	**154**	**-**	**286**	**-**

## Data management and analysis

The data was collected and analysed using Microsoft 2016 Excel (http://www.microsoft.com/en-eg/download/details.aspx?id=50283)

## Discussion

Arboviruses are one amongst the re-emerging pathogens that recently produce unhealthiness everywhere. One-hundred and fifty arboviruses are documented to cause sickness in humans, and the majority is animal diseases, supported by exceeding transmission cycle between arthropods as vectors and vertebrate creature reservoirs as the main amplifying hosts. RVFV is one amongst the foremost aggressive migrating arboviruses. It is transmitted in an enzootic cycle amongst wildlife and mosquitoes, besides wild and farm animals (Figueiredo 2007).

Camel is believed to be resistant to RVFV under clinical conditions, with no apparent infection (Swanepoel & Coetzer 2004). However, clinical signs together with fever and abortion in approximately 10% of pregnant she-camels were determined in free-ranging herds during the 2006–2007 outbreak in Kenya (Munyua et al. 2010). Proof of RVFV circulation amongst domestic camels has also been reported (Munyua et al. 2010; Nabeth et al. 2001; Olaleye, Tomori & Schmitz 1996). The current study revealed that the seroprevalence of RVFV in camels was 21.11%; this result is considered as a high percentage of seropositivity, indicating camels act as reservoir for maintenance of RVFV and high precautions should be applied as camels are used in Egypt for meat and milk production and in Aswan and Luxor for entertainment. The routes of spreading detected to date appear to be in parallel with the migration routes of camels. Therefore, there is some smart proof that viremic, non-symptomatic infected camels transported the virus to Egypt and probably conjointly to the peninsula (Hoogstraal et al. 1979).

Horses appear to be resistant to RVFV (Swanepoel & Coetzer 2004), and experimental infections didn’t result in symptoms or viremia (Daubney & Hudson 1931). However, within the lower Nile River, RVFV antibodies are detected in horses and donkeys (Eisa 1984; Meegan, Hoogstraal & Moussa 1979). Moreover, in Nigeria, RVFV antibodies were detected in horses using a complement fixation test (Olaleye et al. 1989; Olaleye et al. 1996), and the virus was isolated in Nigeria from sheep around 1959; however, this wasn’t related to an epizootic event (Adeyeye, Ekong & Pilau 2011), and serological results in this time that were obtained from zebras (*Equus burchelli*) were negative. This result may clarify that equine might not support RVFV. In this study, the seroprevalence of RVFV in donkey was 0%, indicating that donkeys in Egypt do not maintain RVFV. The result from the examination of cattle, sheep and goat sera revealed that 5 (5.55%), 60 (65.21%) and 13 (14.44%), respectively, were seropositive for RVFV. According to the geographical distribution, Qena had a higher prevalence of RVFV (23.55%) than Aswan (20.65%) and Luxor (14.14%). In regard to the breeding system, there is a higher prevalence of seropositivity in intensive breeding system (27.63%) than individual breeding system (11.68%). The result for farm animals in the southern part of Egypt leads us to indicate that there is no risk factor either from the breeding system or geographic location in the southern part of Egypt. This may be because there is no high difference in temperature and climate in this area.

The geographic distribution of RVF has changed widely from its discovery in 1931 (Daubney & Hudson 1931). Rift Valley Fever disease started in Eastern Africa, then it emerged throughout Africa as well as to Madagascar and the southern part of Egypt. The first step in the control of RVFV disease is early detection of the disease in animals by applying active police roles for investigation and herd monitoring (Davies & Martin 2006). In addition, there is transovarian transmission in arthropod and it is a crucial mechanism for the persistence of RVFV in endemic areas (Linthicum et al. 1988). But transovarian transmission alone is not enough to control of RVFV endemicity over long periods. It should be investigation of different host, including wild animals and domestic mammals, between the large-scale of outbreak.

## Conclusion

This study provides valuable data on the high prevalence of RVFV in sheep and camel in southern Egypt; this will assist in the development of prevention and control strategies for the disease. The high prevalence of RVFV antibodies in camel might be the principal factor limiting the livestock industry in Egypt. More research and effort are needed from governmental and non-governmental authorities to minimise the economic losses caused by RVFV, in addition to the zoonotic risks from RVFV infections.
